# Evaluation of Silver Recovery from High-Sulphur Mining Waste Using Thiourea–Oxalate System

**DOI:** 10.3390/ma18020347

**Published:** 2025-01-14

**Authors:** Erick Jesús Muñoz Hernández, Norman Toro, Martín Reyes Pérez, Iván Alejandro Reyes Domínguez, Aislinn Michelle Teja Ruiz, Mizraim Uriel Flores Guerrero, Jesús Iván Martínez Soto, Gabriel Cisneros Flores, Julio Cesar Juárez Tapia

**Affiliations:** 1 Academic Area of Earth and Materials Sciences, Autonomous University of Hidalgo State, Mineral de la Reforma 42184, Mexico; mu449806@uaeh.edu.mx (E.J.M.H.); mreyes@uaeh.edu.mx (M.R.P.); ma262816@uaeh.edu.mx (J.I.M.S.); ci336682@uaeh.edu.mx (G.C.F.); 2 Faculty of Engineering and Architecture, Universidad Arturo Prat, Iquique 1110939, Chile; notoro@unap.cl; 3 Institute of Metallurgy, Autonomous University of San Luis Potosí, San Luis Potosi 78210, Mexico; alejandro.reyes@uaslp.mx; 4 National Council of Humanities, Sciences and Technologies, Benito Juárez, Mexico City 03940, Mexico; 5 Directorate of Laboratories, Autonomous University of Hidalgo State, Mineral de la Reforma 42184, Mexico; aislinn_teja@uaeh.edu.mx; 6 Industrial Electromechanics Area, Technological University of Tulancingo, Tulancingo 43642, Mexico

**Keywords:** thiourea–oxalate system leaching, mineral tailings, argentite, polybasite, arsenopyrite

## Abstract

Mine tailings are a byproduct of mineral extraction and often pose an environmental challenge due to the contamination of soil and water bodies with dissolved metals. However, this type of waste offers the opportunity for the recovery of valuable metals such as silver (Ag). In the present investigation, an integral analysis of a sample of tailings was carried out, addressing granulometry, elemental composition, neutralization potential (NP), and acid potential (AP), as well as mineralogy, for the dissolution of silver from this type of waste. For this purpose, thiourea (CH_4_N_2_S) was used as a leaching agent due to its low toxicity, and potassium oxalate (K_2_C_2_O_4_) was used as an organic additive to improve the leaching of the silver phases (argentite and polybasite) present in the tailings. The effects of CH_4_N_2_S and K_2_C_2_O_4_ concentrations, temperature, and pH on the leaching efficiency of silver (Ag), copper (Cu), iron (Fe), and arsenic (As) were systematically studied. The results revealed that the maximum silver dissolution rate reached 90.75% under optimal conditions: 0.2 M L^−1^ of thiourea and 0.2 M L^−1^ of potassium oxalate, at 35 °C and a pH of 2.

## 1. Introduction

Tailings are mining residues originating from the ore beneficiation process, where valuable minerals are extracted, leaving behind mineral phases such as iron sulfides, silicates, silica, and minerals containing toxic metals, which are of low economic value [1]. Unfortunately, this type of waste had no environmental regulation during its open-pit disposal, leaving it exposed to weathering factors and microbial activity (e.g., the presence of Thiobacillus ferrooxidans). This exposure results in the oxidation of sulfide-type mineral phases (mainly pyrite), leading to the generation of acid (H^+^) and, as a consequence, the dissolution of heavy metals that accompany the mineral phases of low economic value. The leachates obtained directly contaminate soil and water [2,3,4,5,6,7].

Based on the above, in this type of waste, it is important to evaluate the acid-base balance and the concentration of toxic metals, as well as proposing alternatives that help to totally or partially reduce future acid generation. Due to their content of metals of commercial interest (copper, lead, zinc, gold, and silver), these tailings can also be used as raw material in hydrometallurgical processes for the recovery of valuable metals. Preliminary studies on tailings have shown that controlled leaching processes, under specific conditions (temperatures between 25 °C and 80 °C, reagent concentrations from 0.1 M to 0.5 M, and pH values between 1 and 3), can achieve silver recovery rates between 50% and 85%, depending on the mineralogical composition and the pretreatments applied [5,8,9].

To achieve this objective, it is essential to perform a characterization to identify the mineral phases containing the metals of interest and to analyze the degree of liberation of the metals. In particular, this includes gold and silver, as they are usually found in complex mineral matrices, such as carbonaceous, clayey, arsenosulfide, sulfurized, sulfosalts, and refractory [10,11,12], which means they require specific pretreatments, such as intensive milling, roasting, autoclave oxidation, or bioleaching [13,14]. However, these processes tend to increase operating costs, limiting their economic viability.

On the other hand, it is well known that cyanidation is the most widely used process for the recovery of metals such as gold and silver. However, the environmental problems caused by cyanidation have encouraged the search for new leachants to replace sodium cyanide. Proposed alternatives include chlorides [15], thiosulfate (S_2_O_3_^2−^) [16], thiocyanate (SCN^−^) [17], and thiourea (TU, CS(NH_2_)_2_) [18]. Similarly, oxidants such as manganese dioxide (MnO_2_), monopersulfate compounds (HSO_5_), ozone, hydrogen peroxide (H_2_O_2_), and ferric ion (Fe^3^⁺) have been studied, the latter being the most commonly used in TU leaching. Equation (1) is as follows [19,20,21,22].

(1) $$Ag+2CS({NH}_{2}{)}_{2}+{Fe}^{3+}=Ag(CS({NH}_{2}{)}_{2}{)}_{2}^{+}+{Fe}^{2+}$$

Acid thioureation (TU in acid medium) has proven to be the most attractive alternative for the treatment of silver ores [23] due to its high efficiency and low environmental toxicity [20,24,25]. The use of TU involves the formation of formamidine disulfide (FDS, (NH_2_)_2_CSSC(NH_2_)_2_^2^⁺), as described in Equation (2) [26], which acts as an oxidant in the presence of dissolved oxygen. However, FDS is unstable and decomposes into TU, cyanamide (CH_2_N_2_), and elemental sulfur (S^0^), as shown in Equation (3) [27,28]. The formation of S^0^ is undesirable because it causes mineral surface passivation, which decreases the leaching efficiency of valuable metals [26,29,30].

(2) $$2CS({NH}_{2}{)}_{2}=({NH}_{2}{)}_{2}CSSC({NH}_{2}{)}_{2}^{2+}+{2e}^{-}E{}^{\circ}=0.42\;V$$

(3) $$({NH}_{2}{)}_{2}CSSC({NH}_{2}{)}_{2}=CS({NH}_{2}{)}_{2}+NH_{2}CN+S^{0}$$

According to the study by D. Calla et al. (2016, 2019), TU oxidation is potentiated by an increase in the redox potential (about 0.5 V vs. SHE) caused by the dissolved iron (Fe^3^⁺) and copper (Cu^2^⁺) themselves. Moreover, the ferric ion alone promotes the oxidation of TU, shown in Equation (4), negatively affecting silver leaching [19,31]:

(4) $$2CS({NH}_{2}{)}_{2}+2{Fe}^{3+}=2{Fe}^{2+}+({NH}_{2}{)}_{2}CSSC({NH}_{2}{)}_{2}^{2+}$$

Another negative aspect of the use of thiourea is the ease of forming complexes as metal ions Equations (5)–(7), which implies adding additional TU so as not to mismatch the TU needed in the complex with silver.

(5) $${Fe}^{3+}+CS({NH}_{2}{)}_{2}=[FeCS({NH}_{2}{)}_{2}{]}^{3+}$$

(6) $${Fe}^{3+}+2CS({NH}_{2}{)}_{2}=[Fe(CS({NH}_{2}{)}_{2}{)}_{2}{]}^{3+}$$

(7) $$6CS({NH}_{2}{)}_{2}+2{Cu}^{2+}=2Cu(CS(NH_{2}{)}_{2}{)}_{2}^{+}+{(NH}_{2}{)}_{2}CSSC{(NH}_{2}{)}_{2}^{2+}$$

In attention to the problems of TU oxidation, Chandra et al. (2005) and D. Calla et al. (2020, 2021) have implemented the use of the organic ligand oxalate (Ox, C_2_O_4_^2−^) as a way to decrease the activity of copper and iron ions, Equations (8)–(11), thus allowing for the availability of thiourea for silver dissolution [32,33,34].

(8) $$Cu^{2+}+2C_{2}O_{4}^{2-}=Cu{\left(C_{2}O_{4}\right)}_{2}^{2-}\;\;\;\;\;log\;K=10.23$$

(9) $${Fe}^{3+}+2C_{2}O_{4}^{2-}=Fe{\left(C_{2}O_{4}\right)}_{2}^{-}\;\;\;\;\;log\;K=13.81$$

(10) $${Fe}^{3+}+3C_{2}O_{4}^{2-}=Fe{\left(C_{2}O_{4}\right)}_{3}^{3-}\;\;\;\;\;log\;K=18.6$$

(11) $${Fe}^{3+}+2C_{2}O_{4}^{2-}+2e^{-}=Fe{\left(C_{2}O_{4}\right)}_{2}^{3-}\;\;\;\;\;\;log\;K=16.25$$

Based on the aforementioned issues, this research conducted a characterization and leaching study of mine tailings to evaluate their use as a value-added raw material for silver leaching in a thiourea–oxalate medium. Consequently, this study examines the acid drainage potential through acid–base balance assessments, silver (Ag), copper (Cu), iron (Fe), and arsenic (As) leaching profiles as a function of TU and Ox concentrations, as well as the direct influence of pH and temperature on this leaching process.

## 2. Materials and Methods

### 2.1. Chemical and Mineralogical Characterization

The tailings sample used in this study was provided by the mining company ’El Espiritu’, which is located in the mining district of Zimapan, Hidalgo, Mexico [35]. From the total sample, a quartering process was performed to obtain five kilograms of a representative sample. Subsequently, particle size analysis was conducted using Tyler series sieves, following the methodology outlined by [36].

Each retained fraction was processed through digestion. For this purpose, one gram of each fraction was weighed separately and mixed with 20 mL of aqua regia (HCl and HNO_3_ in a 3:1 ratio) for 60 min. The digestion solutions, along with the samples from the leaching experiments, were analyzed using Inductively Coupled Plasma Optical Emission Spectroscopy (ICP-OES) with a Perkin Elmer Model 8300 instrument. Subsequently, 100 g of the sample was weighed and carefully ground to a particle size of less than 37 μm. This fraction was analyzed by X-Ray fluorescence (XRF) to determine the average composition of the elements present in the sample.

The neutralization potential (NP) was determined using the modified acid–base accounting test (PM-ABA) established in the standard NOM-141-SEMARNAT-2003 [37] and suggested by A. Ruiz-Sánchez [5]. For the determination of NP, one gram of the representative sample was placed on a watch glass, and 0.25 mL of 25% *v*/*v* HCl was added to observe its degree of effervescence (caused by the reaction of calcium carbonate with HCl), which was categorized as none, low, moderate, or strong.

Next, 2 g of the sample was placed in a 250 mL Erlenmeyer flask containing 90 mL of distilled water, and magnetic stirring was set at a speed of 400 rpm. Based on the recorded effervescence level, volumes of 1 N HCl were added at the times indicated in Table 1. After 22 h of stirring, the pH was measured. If the pH was greater than 2.5, additional HCl was added until a pH value of 2 was achieved. However, if the pH measured after 24 h was less than 2, the measurement was repeated due to the possibility of excessive HCl addition at the beginning of the procedure. Finally, the titration was completed with 0.1 N sodium hydroxide (NaOH).

The NP value (g CaCO_3_/kg) was calculated based on the stoichiometry of the reaction in Equation (12), which indicates a proportion of 1.37 g of CaCO_3_ per gram of HCl consumed when neutralizing one kilogram of mine tailings.

(12) $${CaCO}_{3(s)}+2HCL\to {CaCl}_{2}+H_{2}O+CO_{2}$$

The acid potential (AP) was determined based on studies conducted by Petersen, (1969), Edwards et al. (1982), and A. Ruiz-Sanchez et al. (2022) [5,38,39], which approximate AP using the pyrite content in mine tailings through acid digestion. First, 0.2 g of the representative sample was digested with aqua regia (HCl and HNO_3_ in a 3:1 ratio). Subsequently, another 0.2 g of the representative sample was digested with hydrochloric acid (HCl) to remove all iron except that from pyrite. The difference in iron concentration was attributed to the iron from pyrite.

Additionally, 0.2 g was digested with nitric acid (HNO_3_) to dissolve all forms of iron, including pyrite and sulfur. According to the standard [40], the difference in readings between digestion with HNO_3_ and HCl corresponds to pyrite content. The AP value (g CaCO_3_/kg) was determined from the pyrite content and the stoichiometry of the reaction, shown in Equation (13), which indicates a ratio of 1.67 g CaCO_3_ per gram of FeS_2_ for every kilogram of mine tailings.

(13) $$4FeS_{2}+8CaCO_{3}+15O_{2}+{6H}_{2}O\to 4Fe{\left(OH\right)}_{3(s)}+8SO_{4}^{2-}+{8Ca}^{2+}+{8CO}_{2}$$

Characterization of the mine tailings sample was performed using X-Ray Diffraction (XRD) with an EQUINOX 2000 X-Ray diffractometer using Co-Kα1 radiation (1.789010 Å) at 30 mA, 20 kV, and 220 V. The powdered samples were embedded in epoxy resin to form a pellet, which was polished to a mirror finish and analyzed via Scanning Electron Microscopy with Energy Dispersive Spectroscopy (SEM-EDS) using a JEOL JSM-6610LV system. More details on sample characterization can be found in a preliminary study by Erick Muñoz et al., 2023 [41].

### 2.2. Leaching Tests

Leaching experiments were designed to evaluate the dissolution behavior of silver, copper, iron, and arsenic from the tailings under varying conditions.

The following analytical-grade reagents were used for the experiments: thiourea (CH_4_N_2_S, 99%, Sigma-Aldrich, St. Louis, MO, USA), potassium oxalate (K_2_C_2_O_4_, 99.5%, Analytyka, NL, Mexico), Sulfuric Acid (H_2_SO_4_, 98%, Jalmek, NL, Mexico), and Deionized Water (1 μS/cm).

Based on the granulometric and metal content analysis (Section 3.1), mine tailings with a particle size (r_0_) of −53 + 37 μm were selected. Unless otherwise specified, all experiments lasted 60 min. The initial TU and Ox concentrations were based on prior research D. Calla et al. (2016, 2019, 2020, 2021), I. Chandra et al. (2005), Xue-yi Guo et al., 2020 and Erick Muñoz et al., 2022 [19,31,32,33,34,42,43,44]. The reference parameters were set as follows: [TU] = 0.2 M, [Ox] = 0.2 M, 10 g sample, pH = 2, T = 30 °C, and stirring speed = 600 rpm.

The leaching experiments used a Thermo Scientific HP88857190 (MA, USA) heating plate, equipped with a 0.5 L reactor and a stirring system integrated with an IKA EW 20 motor and a Teflon propeller. The pH and temperature values were recorded using a pH/ATC electrode connected to a Thermo Scientific Orion potentiometer.

The pH value (1, 1.5, or 2) was adjusted in 0.5 L of the leaching solution (TU-Ox) before stirring was initiated at a constant speed of 600 rpm (determined in a prior study not included in this work). The temperature was set to 20, 30, or 35 °C. Once the solution reached the target temperature, 20 g of mine tailings were added per liter of leaching solution. The moment the mineral was added was considered the start of the leaching process (t = 0 min). Every 10 min, approximately 10 mL of the leaching liquor was carefully sampled, filtered, and analyzed via ICP-OES to quantify the concentrations of Ag, Cu, Fe, and As.

The leaching percentage was calculated using Equation (14) [45,46]:

(14) $$X_{m}=\frac{C_{E}*V}{M*x}\times 100\%$$

where CE is the concentration of the element measured by ICP-OES (mg/L) in the leaching reactor, V is the total volume of solution (L), x is the mass fraction of the metal (%), and M is the mass of tailings used during leaching (g).

## 3. Results and Discussion

### 3.1. Granulometric Analysis and Metal Content

**Figure 1:**
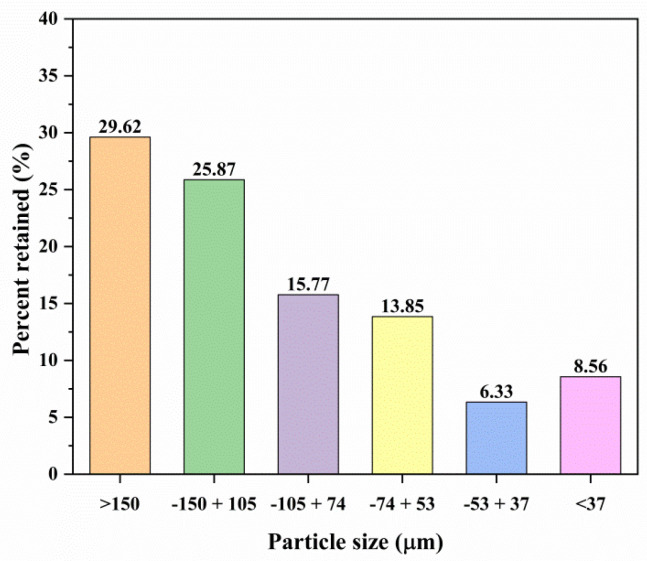
Percentage of mass retained from the tailings of the “El Espiritu” mine.

Figure 1 illustrates the percentage of mine tailings retained on each sieve during the granulometric analysis. As shown, 29.62% of the sample has a particle size greater than 150 μm, while 25.87%, 15.77%, 13.85%, and 6.33% correspond to −150 + 105 μm, −105 + 74 μm, −74 + 53 μm, and −53 + 37 μm, respectively.

Additionally, due to the presence of fine particles smaller than 37 μm (approximately 8.5% of the total sample), it is recommended to securely confine this type of waste. These fine particles pose a risk of being transported by wind, potentially reaching humans through inhalation, which can lead to respiratory diseases such as silicosis [47].

The elemental composition for each particle size range is presented in Table 2. As observed, the silver and copper content tends to increase as the particle size decreases, suggesting that silver phases are more likely to be liberated in smaller particles. Conversely, the composition of Fe and As remains nearly identical for particles within the −150 + 105 μm to −74 + 53 μm size range, with the highest concentration observed in the 37 μm sieve fraction. These results suggest that Fe and As originate from the same mineral phase, most likely arsenopyrite (FeAsS), which is confirmed by SEM in Section 3.3.

The variation in the metal contents shown in Table 2 is evidence that mine tailings are heterogeneous; for this reason, in the leaching tests it is necessary to consider an overall composition ($\%\bar{w}t_{j}$) as a reference to help calculate the leaching percentages. For this, Equation (15), reported in the work of A. Ruiz-Sanchez et al. (2022) [5], was used, where n corresponds to each of the six particle sizes, $\%w_{j}$ is the percentage of mass retained in each fixed particle size n (Figure 1) present in the tailing, and $wt_{j}$ represents the metallic composition for each particle size (Table 2); as a result, an average concentration of 50 g/t, 1.18 Kg/t, 77.60 Kg/t, and 5.28 Kg/t was obtained for Ag, Cu, Fe, and As, respectively.

(15) $$\%\bar{w}t_{j}=\sum_{j=1}^{n}\left(\frac{\%w_{j}}{100})(wt_{j}\right)$$

Another important aspect of the particle size distribution and metallic composition (Table 3) is that about 72% and 52% of the total silver and copper are in particles smaller than 74 μm, so for a hydrometallurgical process of silver dissolution, it is not necessary to perform additional milling. Therefore, it is necessary to consider this type of waste a value-added raw material to establish a complete hydrometallurgical process for silver dissolution.

Table 4 shows the quantitative results obtained by X-ray fluorescence (XRF). One of the advantages of this type of analysis is that it is non-destructive and is carried out quickly. According to the measured concentrations, the main majority elements in the tailings sample are iron (Fe, 303.3 ± 0.6 kg/t), sulfur (S, 292.4 ± 0.4 kg/t), and calcium (Ca, 135.2 ± 0.2 kg/t). On the other hand, the silver (Ag) concentration was 52 ± 3 g/t, similar to the average calculated concentration.

Although the silver leaching process proposed in this study should ideally be conducted using the tailings sample as collected from the deposit site, it is evident that such processing complicates conversion calculations for the target metal. This is because the mass balance would require digesting the entire solid residue after each leaching experiment. Therefore, in the present study, only the −53 + 37 μm fraction was selected, as it contains a nearly uniform silver concentration.

### 3.2. Neutralization Potential (NP) and Acid Potential (AP)

As a result of adding 0.25 mL of 25% *v*/*v* HCl for the NP calculation, a strong effervescence was observed, indicating a significant presence of alkaline species, such as calcite (CaCO_3_) and microcline (Al1.03K0.986Na0.014Si2.97O8), identified in Section 3.3. The pyrite content determined by chemical methods showed comparable results with a minimal difference of approximately 1% (Table 5). This discrepancy could be attributed to the higher aggressiveness of aqua regia, which can dissolve a greater amount of iron, or to the heterogeneity of the sample. Nevertheless, it is important to note that the sample contains at least 8% pyrite (Table 5), which resulted in a calculated acid potential of 140 g CaCO_3_/kg (Table 6). Accordingly, the neutralization potential was determined to be 116.5 g CaCO_3_/kg (Table 6).

The NP/AP ratio was 0.83 (Table 6). According to the standard [37], the tailings are classified as potential acid drainage generators. This highlights the importance of studying metal dissolution from this type of residue and suggests a way to utilize these residues as raw materials. Additionally, the addition of neutralizing agents such as CaCO_3_, Ca(OH)_2_, CaO, Al(OH)_3_, Na_2_CO_3_, and NaOH is recommended to mitigate their environmental impact and enhance their industrial reuse potential [48].

### 3.3. X-Ray Diffraction Analysis (XRD) and Scanning Electron Microscopy Analysis (SEM)

**Figure 2:**
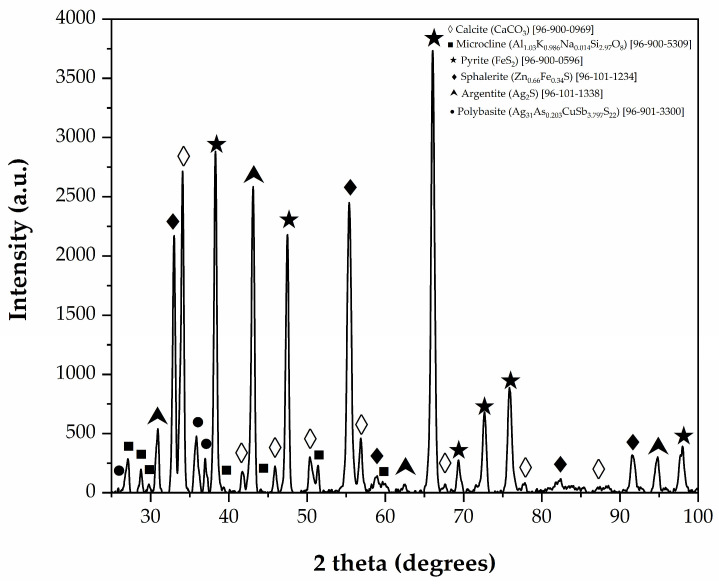
X-ray diffractogram of tailings from the “El Espiritu” mine.

Figure 2 presents the diffractogram of the tailings sample along with the corresponding mineral identification using Match software (version 3). The peaks identified in the diffractogram correspond to calcite [(CaO_3_) (96-101-0963)] and microcline [(Al_1.03_K_0.986_Na_0.014_Si_2.97_O_8_) (96-900-5304)], pyrite [(FeS_2_) (96-901-0596)], sphalerite [(Zn_0.66_Fe_0.34_S) (96-101-1234)], argentite [(Ag_2_S) (96-101-1338)], and polybasite [(Ag_31_As_0.203_CuSb_3.797_S_22_) (96-901-3300)]. These results suggest that silver leaching may not be fully efficient, since part of the silver is present in the form of polybasite, a sulfosalt that is difficult to oxidize [49]. Additionally, it is worth mentioning that the mineral phases identified in this study have also been reported in other research exploring similar tailings [50,51], albeit from a different area within the deposit.

**Figure 3:**
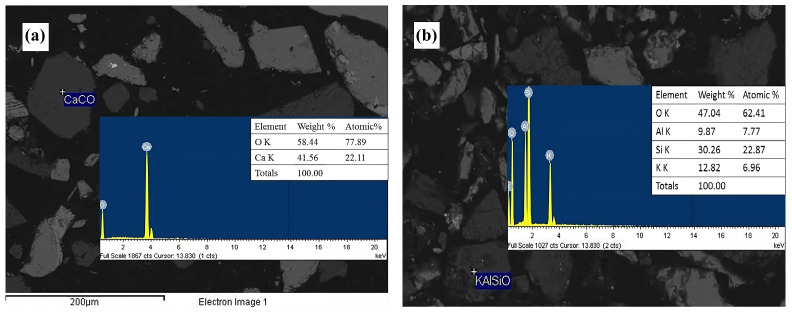
EDS micrographs obtained with backscattered electrons corresponding to the head mineral. (**a**) Calcite (CaCO_3_) and (**b**) feldspar (KAlSi_3_O_8_).

**Figure 4:**
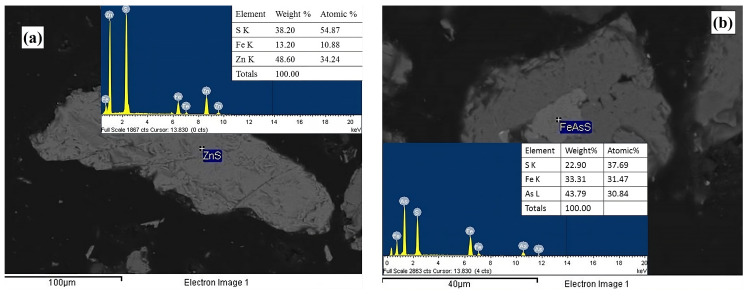
Micrograph and EDS spectra obtained through backscattered electrons of zinc (ZnS) (**a**) and iron (FeAsS) (**b**) species.

**Figure 5:**
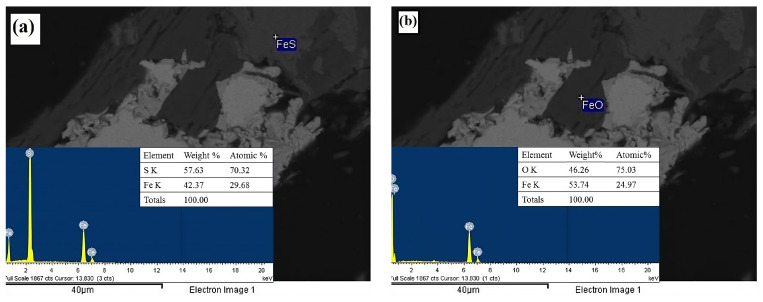
(**a**) Pyrite (FeS2) and (**b**) wustite (FeO). Micrograph obtained through backscattered electron imaging of the mineral species related to iron content.

The spot analyses using energy dispersive spectroscopy (EDS) confirmed the abundant presence of calcite (CaCO_3_, Figure 3a) and feldspar (KAlSi_3_O_8_, Figure 3b), the latter being associated with microcline. This characterization also identified mineral phases such as sphalerite (ZnS, Figure 4a) and arsenopyrite (FeAsS, Figure 4b). EDS analysis further confirmed the presence of pyrite (FeS_2_, Figure 5a) and wüstite (FeO, Figure 5b) as iron-contributing mineral phases. Consequently, the presence of sulfides suggests that pyrite, sphalerite, and arsenopyrite may lead to acid drainage. This occurs because the exposure of these sulfides to moisture and an oxidizing agent, such as atmospheric air, promotes the generation of acid drainage; see Equations (16)–(23) [52,53,54].

(16) $${FeS}_{2}+\frac{7}{2}O_{2}+H_{2}O\to {Fe}^{2+}+2{SO}_{4}^{2-}+2H^{+}$$

(17) $${Fe}^{2+}+\frac{1}{4}O_{2}+H^{+}\to {Fe}^{3+}+\frac{1}{4}H_{2}O$$

(18) $${Fe}^{3+}+3H_{2}O\to {Fe(OH)}_{3(s)}+3H^{+}$$

(19) $${FeS}_{2}+\frac{15}{4}O_{2}+\frac{7}{3}H_{2}O\to {Fe(OH)}_{3(s)}+2{SO}_{4}^{2-}+4H^{+}$$

(20) $$FeAsS+\frac{7}{2}O_{2}+4H_{2}O\to {Fe(OH)}_{3(s)}+{SO}_{4}^{2-}+H_{2}AsO_{4}^{-}+4H^{+}$$

(21) $$4FeAsS+11O_{2}+6H_{2}O\to 4{Fe}^{2+}+4H_{3}AsO_{3}+4{SO}_{4}^{2-}$$

(22) $$2H_{3}AsO_{3}+O_{2}\to 2HAsO_{4}^{2-}+4H^{+}$$

(23) $$2H_{3}AsO_{3}+O_{2}\to 2HAsO_{4}^{-}+4H^{+}$$

If sufficient Fe^3^⁺ ions are present at the deposition site, they act as an oxidizing agent [55], enabling the oxidation of other sulfides, such as sphalerite; see Equation (24) [56]:

(24) $$ZnS+{2Fe}^{3+}\to {Zn}^{2+}+{2Fe}^{2+}+S^{0}$$

**Figure 6:**
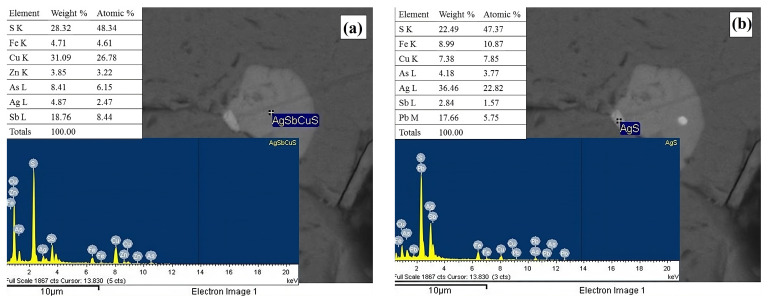
Micrograph obtained by backscattered electrons and EDS spectrum of particles identified as (**a**) polybasite (Ag,Cu)_16_Sb_2_S_11_) and (**b**) argentite (Ag_2_S).

On the other hand, for the mineral phases contributing copper and silver, EDS analyses confirmed the presence of polybasite ((Ag,Cu)_16_Sb_2_S_11_, Figure 6a) and a silver sulfide (argentite, Figure 6b), with both phases previously identified by XRD. However, the micrographs reveal a natural association between polybasite and argentite, similar to that observed with other sulfides such as galena, pyrite, and arsenopyrite [57,58].

### 3.4. Thiourea Concentration

**Figure 7:**
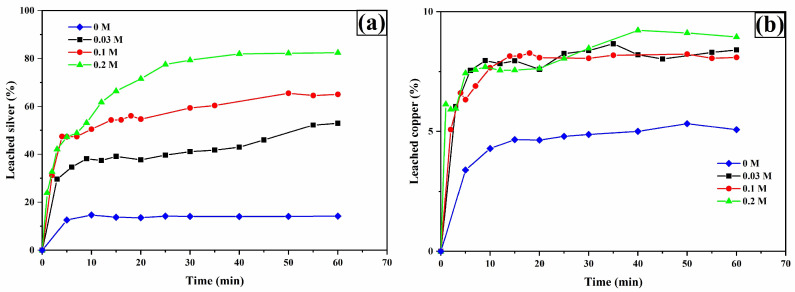
Leaching curves for silver (**a**) and copper (**b**). Experimental conditions: [TU] = 0.2, 0.1, 0.03, and 0 M L^−1^, [Ox] = 0.2 M L^−1^, sample weight = 10 g, pH = 2, temperature = 30 °C, stirring speed = 600 rpm, solution volume = 0.5 L, particle size = −53 + 37 μm.

Figure 7a shows the percentage of silver leaching. As can be seen, the percentage of silver leaching increases with higher TU concentration. The maximum value of silver leaching at 60 min is 52.96%, 65.05%, and 82.40% for 0.03 M, 0.1 M, and 0.2 M, respectively. However, the results show that the presence of TU is important, since in its absence (0 M TU) about 15% is leached.

A tentative explanation for the low percentage of silver leaching with only oxalate is due to the fact that Ag(I)-Ox complexes have lower stability (lower log K values), which means that the presence of Ox^2−^ ligands does not promote the increase in solubility since the only oxidant in the medium is dissolved oxygen, at around 8 mg/L. Instead, the increase in silver leaching rates is due to the fact that Ag(I)-TU complexes are more stable (higher log K) compared to Ag(I)-Ox complexes, as shown in Equations (25)–(27).

In addition, the presence of TU not only helps the stability of Ag(I)-TU complexes, but also promotes the formation of formamidine disulfide ((CS(NH_2_)_2_)_2_^2^⁺), as shown in reactions 28 and 29. The species CS(NH_2_)_2_)_2_^2^⁺ is one more oxidizing species, together with oxygen, that can promote the oxidation of silver, which in the case of Ag_2_S can be represented by the following reactions.

(25) $${Ag}^{+}+{Ox}^{2-}\to Ag({Ox}^{-})$$

(26) $${Ag}^{+}+2CS{\left({NH}_{2}\right)}_{2}\to Ag{{\left(CS{\left({NH}_{2}\right)}_{2}\right)}_{2}}^{+}$$

(27) $${Ag}^{+}+3CS{\left({NH}_{2}\right)}_{2}\to Ag{{\left(CS{\left({NH}_{2}\right)}_{2}\right)}_{3}}^{+}$$

(28) $$CS{\left({NH}_{2}\right)}_{2}+H^{+}\to {HCS{\left({NH}_{2}\right)}_{2}}^{+}$$

(29) $${2HCS{\left({NH}_{2}\right)}_{2}}^{+}\to {{\left(CS{\left({NH}_{2}\right)}_{2}\right)}_{2}}^{+}+{2H}^{+}+{2e}^{-}$$

(30) $${Ag}_{2}S+6CS{\left(NH_{2}\right)}_{2}+{\frac{1}{2}O}_{2(ac)}+{2H}^{+}↔2Ag{{\left[\left(CS{\left(NH_{2}\right)}_{2}\right)\right]}_{3}}^{+}+S^{0}+H_{2}O$$

(31) $${Ag}_{2}S+4CS{\left(NH_{2}\right)}_{2}+{{\left(CS{\left({NH}_{2}\right)}_{2}\right)}_{2}}^{+}↔2Ag{{\left[\left(CS{\left(NH_{2}\right)}_{2}\right)\right]}_{3}}^{+}{+S}^{0}$$

**Figure 8:**
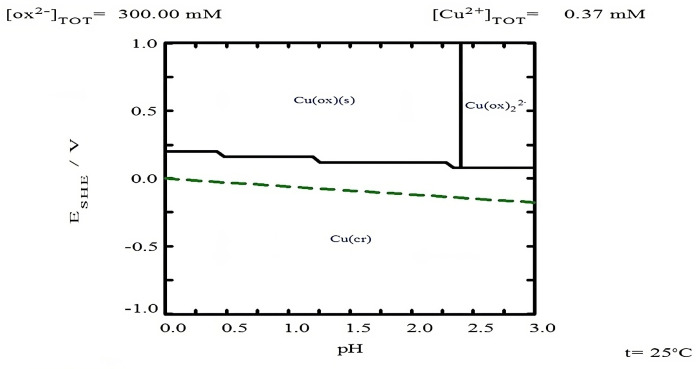
Eh-pH diagram for [Cu^2^⁺] = 3.726 × 10^−^⁴ M with 0.3 M oxalate at 25 °C. Diagram generated using MEDUSA© software.

As for copper (Figure 7b), it is observed that in the absence of TU, the percentage of copper leaching was around 5.22% at 20 min. This percentage does not increase, probably because equilibrium is reached in the leaching solution for the formation of copper oxalate, as shown in the Pourbaix-type diagram in Figure 8, which was created with the free version of MEDUSA© software (Making Equilibrium Diagrams Using Simple Algorithms), which can be downloaded from the following page https://www.kth.se/che/medusa/downloads-1.386254 (accessed on 1 January 2025) [59], and described by Equation (32).

The increase of at least 5% more in silver dissolution can be attributed to the formation of TU-Cu(II) complexes that promote the solubility of copper in the form of solid copper oxalate. Meanwhile, the increase in copper solubility from solid copper oxalate is not as significant due to the higher log K Equation (33) for the Ox-Cu(II) complexes compared to the TU-Cu(II) complexes, whose thermodynamic constants are shown in Equations (34)–(37) [60,61].

(32) $${Cu}^{2+}+{Ox}^{2-}\to Cu{\left(Ox\right)}_{\left(s\right)}\;\;\;\;\;\;log\;K=11.19$$

(33) $${Cu}^{2+}+{2Ox}^{2-}\to Cu{{\left(Ox\right)}_{2}}^{2-}\;\;\;\;\;\;log\;K=10.23$$

(34) $${Cu}^{2+}+CS{\left({NH}_{2}\right)}_{2}\to Cu{\left(CS{\left({NH}_{2}\right)}_{2}\right)}^{2+}\;\;\;\;\;log\;K=0.8$$

(35) $${Cu}^{2+}+2CS{\left({NH}_{2}\right)}_{2}\to Cu{{\left(CS{\left({NH}_{2}\right)}_{2}\right)}_{2}}^{2+}\;\;\;\;\;log\;K=0.8$$

(36) $${Cu}^{2+}+3CS{\left({NH}_{2}\right)}_{2}\to Cu{{\left(CS{\left({NH}_{2}\right)}_{2}\right)}_{3}}^{2+}\;\;\;\;\;\;log\;K=0.9$$

(37) $${Cu}^{2+}+4CS{\left({NH}_{2}\right)}_{2}\to Cu{{\left(CS{\left({NH}_{2}\right)}_{2}\right)}_{4}}^{2+}\;\;\;\;\;\;log\;K=1.1$$

**Figure 9:**
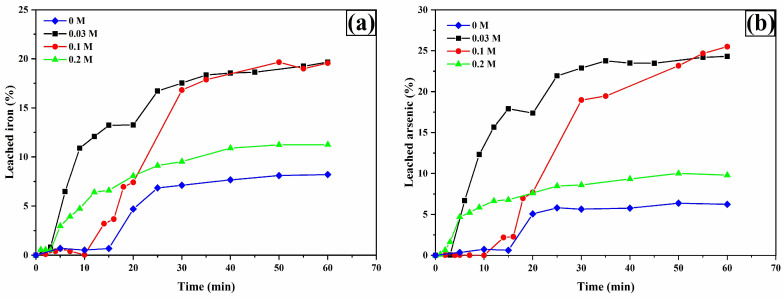
Leaching curves for iron (**a**) and arsenic (**b**). Experimental conditions: [TU] = 0.2, 0.1, 0.03, and 0 M L^−1^, [Ox] = 0.2 M L^−1^, sample: 10 g, pH = 2, T = 30 °C, stirring speed = 600 rpm, solution volume = 0.5 L, particle size = −53 + 37 μm.

The analysis of iron (Figure 9a) and arsenic (Figure 9b) leaching shows similar behaviors, suggesting that the leaching process for these metals originates from the oxidation of arsenopyrite. This is deduced from the dissolution percentage ratios of Fe and As at 60 min in the presence of 0.2 M TU, with values of 19.44% and 25.68% (As/Fe ratio of 1.32), which is numerically close to the 1.34 ratio derived from the molecular weights of As and Fe in the arsenopyrite chemical formula.

On the other hand, the behavior during the first 20 min of Fe and As leaching percentages does not show a clear trend, possibly due to sample heterogeneity, the natural association of arsenopyrite with other mineral phases, or the occlusion of arsenopyrite within other mineral phases present in the tailings. However, it is evident that TU presence promotes arsenopyrite dissolution, as oxalate stabilizes thiourea, preventing its oxidation by arsenopyrite itself or by the Fe (III) resulting from arsenopyrite oxidation, as suggested by Ke Li et al. (2024) in their study on arsenopyrite oxidation using Fe (III) ions with thiourea. Based on Ke Li et al.’s work, the arsenopyrite leaching process can be described by reaction 38 [62].

(38) $$FeAsS+{\frac{3}{2}O}_{2(ac)}+{2Ox}^{2-}↔{Fe{\left(Ox\right)}_{2}}^{-}+{{AsO}_{3}}^{3-}{+S}^{0}$$

### 3.5. Oxalate Concentration

**Figure 10:**
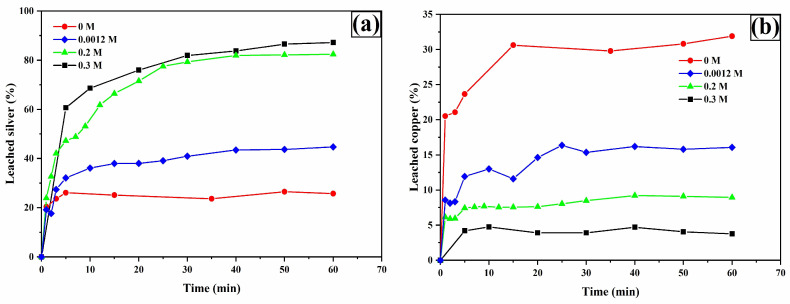
Leaching curves for silver (**a**) and copper (**b**). Experimental conditions: [TU] = 0.2 M L^−1^, [Ox] = 0.3, 0.2, 0.0012, and 0 M L^−1^, sample: 10 g, pH = 2, T = 30 °C, stirring speed = 600 rpm, solution volume = 0.5 L, particle size = −53 + 37 μm.

To evaluate the impact of oxalate ion concentration on the leaching of Ag, Cu, Fe, and As, the TU concentration was fixed at 0.2 M, along with the parameters defined in the methodology (see Section 2.2: Leaching System). Figure 10a shows the silver percentages obtained with and without the presence of oxalate. In the absence of oxalate, the maximum silver dissolution percentage reached was 25.77%. However, it is evident that the presence of oxalate, ranging from 0.0012 M to 0.3 M L^−1^, promotes an increase in silver leaching percentages up to 87.70%.

The benefit of using oxalate in silver leaching is that it prevents the decomposition of TU by arsenopyrite. In the absence of oxalate, as reported by Ke Li et al. (2024), arsenopyrite catalyzes the decomposition of thiourea into formamidine disulfide, an unstable species that easily decomposes. Consequently, TU is depleted, and the leaching process reaches equilibrium within 5 min [62].

**Figure 11:**
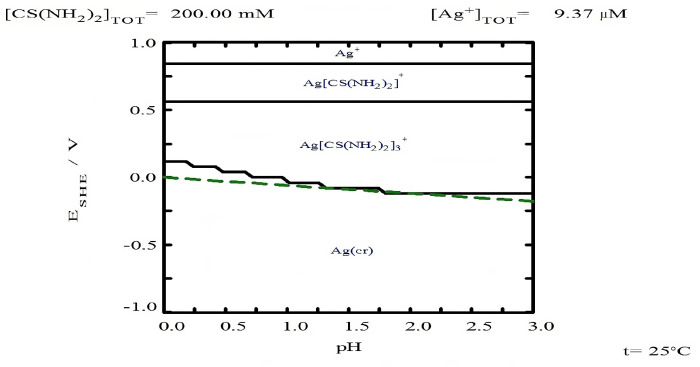
Eh-pH diagram for [Ag] 9.7 × 10^−^⁶ M with 0.2 M thiourea at 25 °C. Diagram generated using MEDUSA© software.

**Figure 12:**
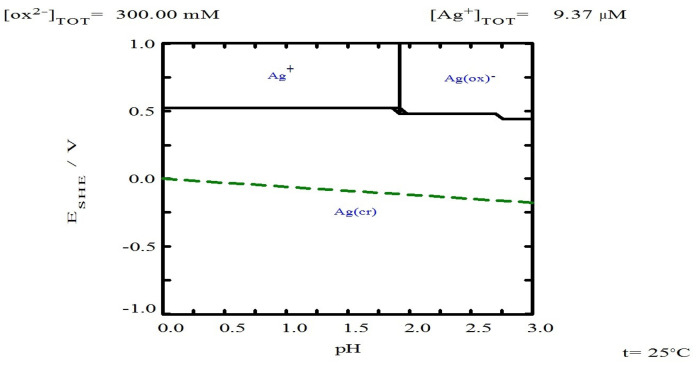
Eh-pH diagram for [Ag] 9.37 × 10^−^⁶ M with 0.3 M oxalate at 25 °C. Diagram generated using MEDUSA© software.

In addition to the above analysis, it is also observed that the increase in the oxalate concentration from 0.2 M to 0.3 M does not significantly benefit the dissolution of silver in the form of Ag(CS(NH_2_)_2_)_3_^+^. This is probably due to the fact that the Ag(I)-Tu complexes have a larger solubility window in terms of potential and pH, as shown in the Pourbaix diagram (Figure 11). This behavior is not observed for the Ag(I)-Ox system, where the formation of the Ag(Ox)⁺ complex is limited by the maximum pH value reached in the experiments (Figure 12). 

It should be noted that Figure 11 and Figure 12 were created using the free version of the MEDUSA© software (Making Equilibrium Diagrams Using Simple Algorithms), which can be downloaded from the following page: https://www.kth.se/che/medusa/downloads-1.386254 (accessed on 1 January 2025) [59]. Additionally, the MEDUSA© software does not contain thermodynamic data related to TU species; therefore, these data were collected from references and incorporated using the advanced method, as detailed in Appendix A.

According to Figure 11 and Figure 12, both TU and oxalate ions can form complexes with silver. This suggests a synergistic interaction that explains the improved leaching percentages within a shorter time frame. This complex interaction between silver, thiourea, and oxalate suggests it has also been reported by Xiyun Yang et al. (2011) [63] when using thiourea with thiocyanate. For the above mentioned information, it is thought that the best leaching conditions are the use of 0.2 M of Tu and Ox, respectively.

The copper leaching profiles (Figure 10b) show that in the absence of oxalate ions, the maximum copper concentration (30.63%) is achieved, which simultaneously implies the maximum dissolution of silver associated with copper, specifically from polybasite. Given that the Ag/Cu mass ratio in polybasite is approximately 1.64, it can be inferred that about 50% of the total leached silver (87.7%) originates from polybasite. Additionally, it is observed that increasing the oxalate concentration to 0.3 M results in a decrease in dissolved copper due to the formation of solid copper oxalate (Figure 8), as demonstrated by Ruiz-Sánchez et al. (2020) in the chalcopyrite leaching process using organic ligands [64].

Consequently, the competition between Cu ions and Ag ions for the formation of complexes with thiourea is reduced.

**Figure 13:**
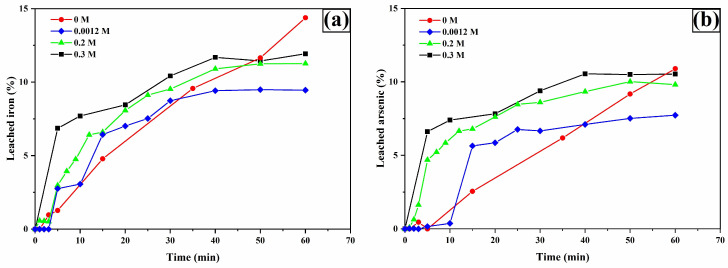
Leaching curves for iron (**a**) and arsenic (**b**). Experimental conditions: [TU] = 0.2 M L^−1^, [Ox] = 0.3, 0.2, 0.0012, and 0 M L^−1^, sample = 10 g, pH = 2, T = 30 °C, stirring speed = 600 rpm, solution volume = 0.5 L, particle size = −53 + 37 μm.

Figure 13a,b show leaching profiles for iron and arsenic with similar trends and relationships to the results in Figure 9, supporting the assertion that these metals in aqueous form originate from arsenopyrite oxidation. Interestingly, in the absence of oxalate, the dissolution of copper and iron follows an almost linear trend, suggesting rapid and preferential dissolution of arsenopyrite. This behavior indicates that, without oxalate ions, arsenopyrite catalyzes the decomposition of thiourea into formamidine disulfide, an unstable species that leads to cyanamide (CH_2_N_2_) and elemental sulfur (S^0^) [27]. Consequently, the silver phases present are not significantly oxidized, resulting in a silver recovery of only about 20% (Figure 10).

### 3.6. pH Effect

**Figure 14:**
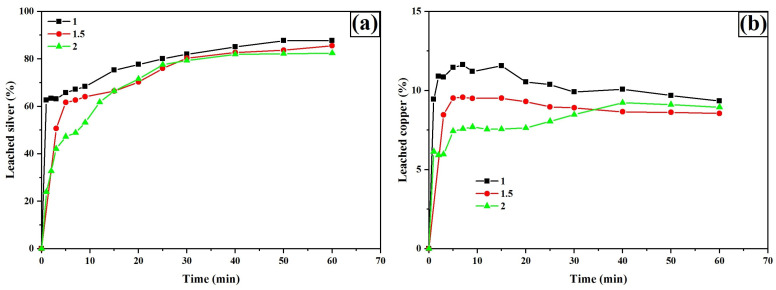
Leaching curves for silver (**a**) and copper (**b**). Experimental conditions: [TU] = 0.2 M L^−1^, [oxalate] = 0.2 M L^−1^, sample = 10 g, pH = 1, 1.5, and 2, T = 30 °C, stirring speed = 600 rpm, solution volume = 0.5 L, particle size = −53 + 37 μm.

Figure 14a presents silver leaching percentages as a function of the initial pH of the leaching solution. The results indicate that acidic pH levels enhance the dissolution of silver phases, achieving 88%, 87%, and 86% silver recovery within the first 5 min for pH values of 1, 1.5, and 2, respectively. This behavior is attributed to the precipitation of copper as copper oxalate (Figure 14b) on the surface of silver phases at pH = 1, which creates a transport resistance for reactants toward the reaction surface. This phenomenon is reflected in the slower increase in silver leaching profiles for pH = 1 and 1.5 during the first 5 min.

**Figure 15:**
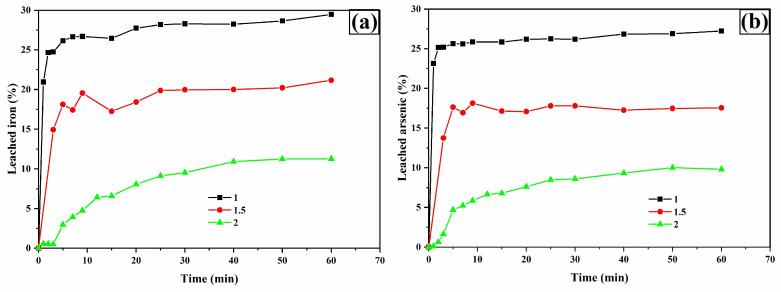
Leaching curves for iron (**a**) and arsenic (**b**). Experimental conditions: [TU] = 0.2 M L^−1^, [oxalate] = 0.2 M L^−1^, sample = 10 g, pH = 1, 1.5, and 2, T = 30 °C, stirring speed = 600 rpm, solution volume = 0.5 L, particle size = −53 + 37 μm.

**Figure 16:**
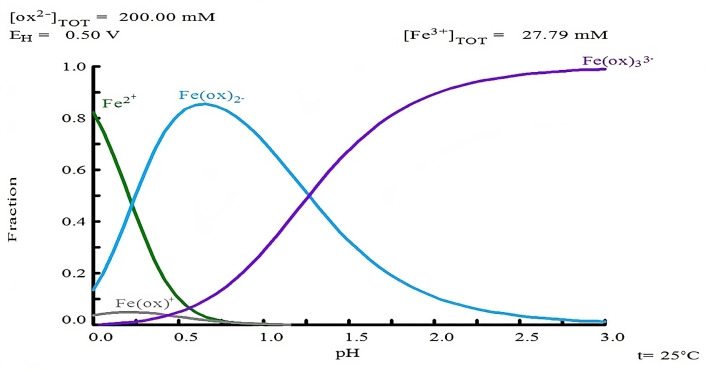
Species distribution diagram for Fe with 0.2 M oxalate as a function of pH. Conditions: 0.02779 M Fe and 0.2 M oxalate at 25 °C. Diagram generated using MEDUSA© software [59].

Iron (Figure 15a) and arsenic (Figure 15b) leaching experienced a notable increase as the pH decreased. For Fe, leaching percentages of 29.48% and 21.16% were observed at pH = 1 and 1.5, respectively. In the case of As, leaching percentages reached 27.23% and 17.55%. Lowering the pH triggers significant changes in species chemistry, favoring the formation of soluble species such as Fe(Ox)^+^, Fe(Ox)_2_^−^ and Fe(Ox)_3_^3−^, as illustrated in the species distribution diagram (Figure 16).

### 3.7. Temperature Effect

**Figure 17:**
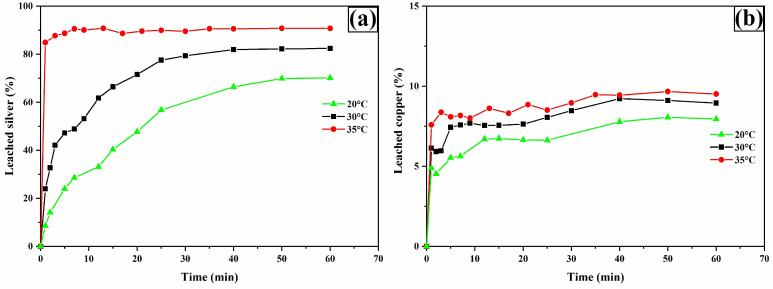
Leaching curves for silver (**a**) and copper (**b**). Experimental conditions: [TU] = 0.2 M L^−1^, [Ox] = 0.2 M L^−1^, sample weight = 10 g, pH = 2, temperatures = 20, 30, and 35 °C, stirring speed = 600 rpm, solution volume = 0.5 L, particle size = −53 + 37 μm.

Figure 17a shows silver leaching profiles as a function of temperature. The results indicate that increasing the temperature enhances the dissolution of silver phases. This effect suggests chemical reaction control, as raising the temperature from 20 °C to 35 °C allows for the recovery of 90% (around 0.105 g/L) of the available silver. Additionally, it is observed that higher temperatures accelerate equilibrium attainment, requiring only 5 min of operation at 35 °C. This effect implies that higher temperatures improve the dissolution kinetics of silver, but simultaneously promote the rapid decomposition of thiourea, preventing the leaching of the remaining 10% silver. In fact, regardless of the operating temperature, it was found that after 45 min, thiourea concentration had decreased by more than 70% from its initial value (0.2 M), which was already in excess relative to the stoichiometry of reactions 31 and 32.

**Figure 18:**
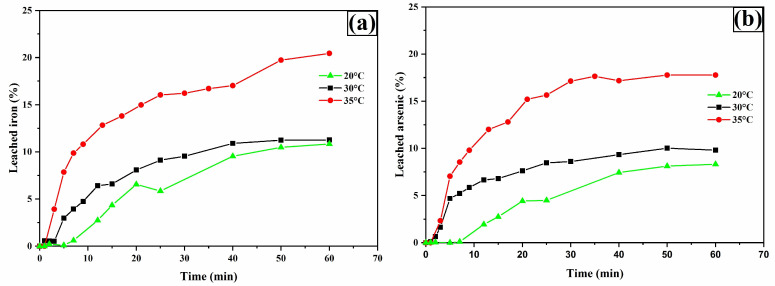
Leaching curves for iron (**a**) and arsenic (**b**). Experimental conditions: [TU] = 0.2 M L^−1^, [Ox] = 0.2 M L^−1^, sample weight = 10 g, pH = 2, temperatures = 20, 30, and 35 °C, stirring speed = 600 rpm, solution volume = 0.5 L, particle size = −53 + 37 μm.

The leaching profiles for copper (Figure 17b), iron, and arsenic (Figure 18a,b) were also enhanced by increasing the temperature.

## 4. Conclusions

- The TU-Ox system represents a suitable option for recovering precious metals present in tailings. This approach adds value to these residues, which are rich in sulfides and may potentially generate acid drainage in the future.
- The most important variables that help to optimize this process are the temperature and concentration of thiourea and oxalate. The optimum conditions were 0.2 M thiourea, 0.2 M potassium oxalate, pH=2 and 35 °C, and a maximum silver recovery of 90.75% was achieved.
- The TU-Ox system demonstrated improvements in the leaching process, particularly in the presence of arsenopyrite, a mineral phase that catalyzes the decomposition of thiourea.
- Oxalate helps stabilize thiourea, ensuring its availability to form complexes with silver. However, increasing oxalate concentration can lead to the formation of solid copper oxalate, which creates resistance to the transport of reagents and products.
- Higher temperatures enhance silver leaching, suggesting that the process is chemically controlled. However, elevated temperatures also accelerate thiourea decomposition.

## Figures and Tables

**Table 1:** HCl addition for NP determination.

Effervescence Degree (Carbonate Neutralization)	HCl 1 N Volume (mL)	
	0 h	24 h
None	1	1
Low	2	1
Moderate	2	2
Strong	3	2

**Table 2:** Elemental composition of tailings at different particle sizes.

Particle Size (μm)	Elemental Composition (Kg/t)			
	Ag *	Cu	Fe	As
>150	30	1.13	65.69	2.52
−150 + 105	20	1.01	78.60	4.39
−105 + 74	20	0.82	78.51	4.81
−74 + 53	70	0.45	68.37	8.60
−53 + 37	120	2.65	152.38	13.05
<37	180	2.68	73.75	7.33

* Composition expressed in g/t.

**Table 3:** Distribution of Ag, Cu, Fe, and As content at different particle sizes.

Particle Size (μm)	(%)			
	Ag	Cu	Fe	As
>150	17.06	28.18	25.07	14.10
−150 + 105	10.91	21.99	26.20	21.49
−105 + 74	6.03	10.98	15.95	14.34
−74 + 53	20.54	5.32	12.20	22.55
−53 + 37	15.39	14.18	12.43	15.63
<37	30.08	19.36	8.14	11.88

**Table 4:** Majority and minority elements present in Jal.

Element	Concentration (Kg/t)	Element	Concentration (g/t)
Fe	303.3 ± 0.6	Ti	475 ± 2
S	292.4 ± 0.4	Cd	360 ± 51
Ca	135.2 ± 0.2	Co	241 ± 87
Mg	52.9 ± 0.7	Sb	178 ± 6
Zn	50.6 ± 0.2	Zr	94 ± 5
Si	46.9 ± 0.4	Sr	73 ± 4
Al	14.9 ± 0.6	Ag	52 ± 3
Pb	12.9 ± 0.1	Hg	32 ± 11
As	10.5 ± 0.1		
K	3.85 ± 0.5		
Mn	2.86 ± 0.8		
Cu	1.36 ± 0.6		

**Table 5:** Pyrite content analysis in tailings using chemical methods.

Iron Content%			
Aqua regia	HCL	Pyritic iron	Pyrite content (%)
4.3	0.6	3.8	8.1
HNO3	HCL	Pyritic iron	Pyrite content (%)
3.9	0.6	3.3	7.1

**Table 6:** Neutralization potential and acid potential relationship.

Parameter	Value
NP (g CaCO_3_/kg)	116.5
AP (g CaCO_3_/kg) *	140
Relación NP/AP	0.83

* Estimation considering 8.1% FeS_2_ obtained by chemical method.

## Data Availability

The original contributions presented in the study are included in the article; further inquiries can be directed to the corresponding author.

## Appendix A

The following equilibrium constants (Log K) for the relevant redox reactions and complexes, used in the MEDUSA program at 25 °C with thiourea and Ag, are presented in [29].

(A1) $${Ag}^{+}+CS(N{H_{2})}_{2}=Ag[CS(N{{H_{2})}_{2}]}^{+}\;\;\;log\;K=7.59$$

(A2) $${Ag}^{+}+2CS(N{H_{2})}_{2}=Ag[CS(N{{H_{2})}_{2}{]}_{2}}^{+}\;\;\;log\;K=10.35$$

(A3) $${Ag}^{+}+3CS(N{H_{2})}_{2}=Ag[CS(N{{H_{2})}_{2}{]}_{3}}^{+}\;\;\;log\;K=12.87$$

(A4) $${Ag}^{+}+4CS(N{H_{2})}_{2}=Ag[CS(N{{H_{2})}_{2}{]}_{4}}^{+}\;\;\;log\;K=13.57$$

(A5) $${Ag}^{+}+3CS(N{H_{2})}_{2}={Ag}_{2}[CS(N{{H_{2})}_{2}{]}_{3}}^{+}\;\;\;log\;K=20.70$$
